# Docetaxel- and 5-FU-concurrent radiotherapy in patients presenting unresectable locally advanced pancreatic cancer: a FNCLCC-ACCORD/0201 randomized phase II trial's pre-planned analysis and case report of a 5.5-year disease-free survival

**DOI:** 10.1186/1748-717X-6-124

**Published:** 2011-09-26

**Authors:** Lucie Oberic, Frédéric Viret, Charlotte Baey, Marc Ychou, Jaafar Bennouna, Antoine Adenis, Didier Peiffert, Françoise Mornex, Jean-Pierre Pignon, Patrice Celier, Jocelyne Berille, Michel Ducreux

**Affiliations:** 1 Institut Gustave Roussy, Villejuif, France; 2 Institut Paoli Calmettes, Marseille, France; 3 Centre Val d'Aurelle, Montpellier, France; 4 Centre René Gauducheau, Nantes, France; 5 Centre Oscar Lambret, Lille, France; 6 Centre Alexis Vautrin, Nancy, France; 7 Hôpital Lyon Sud, Lyon, France; 8 Centre Paul Papin, Angers, France; 9 FNCLCC, Paris, France; 10 Université Paris Sud 11, Le Kremlin Bicetre, France

## Abstract

**Background:**

To explore possible improvement in the treatment of locally advanced pancreatic carcinoma (LAPC) we performed a randomized, non-comparative phase II study evaluating docetaxel - plus either daily continuous 5 FU or weekly cisplatin concurrent to radiotherapy. We report here the results of the docetaxel plus 5 FU regimen stopped according to the interim analysis. The docetaxel plus cisplatin arm was continued.

**Methods:**

Forty (40) chemotherapy-naive patients with unresectable LAPC were randomly assigned (1:1) to either continuous fluorouracil (5-FU) 200 mg/m^2^/day (protracted IV) and docetaxel (DCT) 20 mg/m^2^/week or DCT 20 mg/m^2 ^and cisplatin (CDDP) 20 mg/m^2^, plus concurrent radiotherapy for a period of 6 weeks. The radiation dose to the primary tumor was 54 Gy in 30 fractions. The trial's primary endpoint was the 6-month crude non-progression rate (NPR). Secondary endpoints were tolerance, objective response rate, and overall survival. Accrual was to be stopped if at 6 months more than 13 disease progressions were observed in 20 patients.

**Results:**

Eighteen (18) progressions occurred at 6 months in the 5-FU-DCT arm. Six-month NPR was 10% (95%CI: 0-23). Six and 12-month survivals were 85% (95%CI: 64-95) and 40% (95%CI: 22-61); median overall survival was 10.1 months. Median progression-free survival was 4.3 months. We report the case of one patient who was amenable to surgery and has been in complete response (CR) for 5.5 years. Toxicities grade ≥ 3 were reported in 75% of patients; no treatment-related death occurred. Severe toxicities were mainly vomiting (35%), abdominal pain (10%) and fatigue (10%).

**Conclusions:**

Combination of 5-FU, docetaxel and radiotherapy has inadequate efficacy in the treatment of LAPC despite good tolerance for the 5-FU-DCT regimen.

**Trial Registration:**

ClinicalTrials.gov: NCT00112697

## Background

Pancreatic cancer (PC) is an extremely aggressive malignancy and the 4^th ^cause of all cancer deaths worldwide [1]. Unfortunately, because of the typically late onset of symptoms and the persistent lack of early detection, the rate of PC cases amenable to surgical resection at the time of diagnosis has remained unchanged, around (15%-20%), over the past decades [2]. More than 50% of patients with PC are unresectable because of the metastatic spread of the disease at initial presentation, and the remaining 30% unresectable are due to local extension with vascular involvement [3]. Overall, the acknowledged 5-year survival rate for exocrine pancreas adenocarcinoma is around 3% - 5% [4,5]. In case of loco-regional disease development, survival is relatively better. However, with a median survival of only 6 to 8 months the patient's chances of surviving several years remain low. About 10%-15% of resected patients survive more than 5 years and less than 5% more than 10 years [5,6].

Compared to radiotherapy alone, 5-FU concurrent radiotherapy has become a widespread standard that can be used in locally advanced PC, either pre- or post-operatively [7]. In the pre-operative setting, chemoradiation is used to gain locoregional control in the treatment of border line resectable cancer [8]. Chemoradiation facilitates or makes the resection possible, especially when the tumor is too large or if it makes contact with the vascular system. Post-operative chemoradiation is used to improve survival [9]. Although there is no definite evidence of the superiority of either its tolerance or efficacy compared to bolus 5-FU, continuous (protracted) 5-FU intravenous infusion, delivered with concurrent radiotherapy (RT), is of common use in the treatment of a number of gastrointestinal cancers including pancreatic and colorectal carcinoma [10,11]. Continuous infusion insures a more constant concentration of radio-sensitizing agent at the tumor site throughout the period of radiotherapy. Although 5-FU-based chemoradiation has an acceptable response rate (20%) and a low toxicity, the ideal schedule has not yet been established [12]. Docetaxel (DCT) is a semisynthetic taxane with a large spectrum of antitumoral activity including pancreatic cancer [13]. The activity of this drug in first-line metastatic patients has been demonstrated as has its radiosensitizing potential [14-16]. Several phase II and phase III trials have shown that the addition of both cisplatin and fluorouracil to docetaxel did not increase toxicity [17].

The Federation Nationale des Centres de Lutte contre le Cancer (FNCLCC) has designed this randomized phase II study to explore the possibility of combining DCT with either cisplatin or 5-FU to improve concurrent chemo- and radiation therapy in the treatment of non resectable LAPC. We report here the study arm where docetaxel was combined to 5-FU and briefly discuss a long-term survival case.

## Methods

Patients participating in this non-comparative, multicenter, phase II study were centrally randomized at the Gustave-Roussy Institute in Villejuif, France using minimization on center, performance status and age. An interim analysis was planned after inclusion of 20 patients in each arm. The results we report here are only for the 5-FU-docetaxel concurrent radiotherapy arm that was discontinued after the interim analysis. This research was carried out in compliance with the Helsinki Declaration. The protocol was approved by the Ethical Committee of Kremlin-Bicêtre, the review committees of the FNCLCC and the participating institutions. Trial registration: Current Controlled Trials NCT00112697;

### Patient Population

Patients of age > 18 years and < 75 years with pathologically confirmed unresectable locoregional advanced adenocarcinoma of the exocrine pancreas were eligible. Other histological subtype of pancreatic tumors including neuroendocrine tumor and ampulla of Vater were not eligible. Unresectability was defined by a surgeon and evaluated after laparotomy or according to CT-scan and/or endoscopic criteria, including vascular involvement. Measurable disease was required. Patients with clinical or radiologic diagnosis of metastases were excluded. No prior chemotherapy was allowed; patients were required to have a Karnofsky performance status (PS) higher than 70, adequate baseline bone marrow function (i.e., neutrophiles count > 1,500/μL and platelets > 100,000/μL), normal serum creatinine levels (< 120 μmol/l), and bilirubin levels < 1.5 times the upper limit of normal (ULN) after biliary drainage. Patients with prior history of another primary tumor within the last 10 years, except adequately treated *in situ *carcinoma of the cervix uteri and basal or squamous cell skin carcinomas, and patients with grade II peripheral neuropathy according to NCI-CTC criteria were excluded. Written informed consent was obtained according to the French regulations.

### Treatment Plan

**Figure 1:**
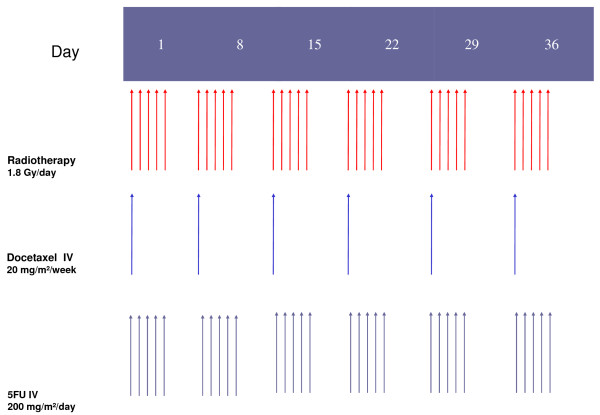
**Treatment schedule: 5-FU + docetaxel + radiotherapy**.

Patients were randomly assigned to receive continuous 5-FU 200 mg/m^2^/day and docetaxel (DCT) 20 mg/m^2^/week (Figure 1) or weekly docetaxel and cisplatin. Treatment was administered for at least six weeks unless disease progression was documented, unacceptable toxicity, or patient refusal occurred. Premedication included adequate antiemetic therapy, dexamethasone (IV) before each docetaxel infusion and prophylactic granulocyte colony-stimulating factor (C-GSF) treatment in case of severe hematotoxicity.

Dose adjustments were based on the worst toxicity observed during the previous cycle. Radiotherapy consisted of an initial 54 Gy in 30 fractions (dose specified at the isocenter) with minimum photon energy of 6 MeV. The initial field covered the gross tumor volume and regional nodes, including the celiac axis.

### Response and Toxicity Evaluation

Because evaluating tumor response in LAPC is difficult, we used progression instead of objective response as the main study endpoint. Progressive disease (PD) was defined as the appearance of a metastasis or protein-rich ascites or duodenal stenosis, and/or an increase > 30% of the lesion size calculated as the sum of the two longest perpendicular diameters of the tumor. Tumors were evaluated with the modified RECIST scale at week 12 (i.e. 5 weeks after the treatment's end) and every 2 months thereafter until disease progression or patient's death. Toxicities were graded according to the NCICTC scale (version 2.0) for chemotherapy, and according to the RTOG criteria for radiotherapy.

### Statistical Design

The primary endpoint was the 6-month crude non-progression rate (NPR). All eligible patients who initiated study treatment were included in the primary endpoint analysis. Considering a 35% control rate, the trial power to detect a 6-month crude NPR of 60% was 93% with a type I error of 4% based on a Fleming design [18]. An interim analysis was planned after the first 20 enrolled patients experienced the treatment protocol for 6 months. Enrollment was to be continued with 20 additional patients if between 8 and 12 cases of non-progression were observed at 6 months. With 7 patients or less, the regimen would be considered non efficient and the corresponding arm terminated. Conversely with more than 13 out of 20 patients exhibiting non progressive diseases, the experimental arm would therefore be considered as active and stopped. Secondary endpoints included adverse events, progression-free survival (PFS), overall survival (OS), and objective response (OR) according to the RECIST criteria. Adverse events were evaluated before each cycle of treatment. Overall and progression-free survivals were estimated using the Kaplan-Meier method. Overall survival was defined as the time from randomization to the date of death or to the date of lost follow-up and progression-free survival as the time from randomization to disease progression or death, whatever its cause, or last follow-up.

Written informed consent was obtained from the patient for publication of this case report and accompanying images. A copy of the written consent is available for review by the Editor-in-Chief of this journal.'

## Results

Results are reported only for arm A which was closed prematurely, i.e. continuous infusion of 5-FU plus docetaxel and radiotherapy.

### Patient characteristics

Between November 2003, and August 2005, 20 patients were included in 6 centers. Patient characteristics are listed in Table 1. Median age was 62 years, and all patients had PS between 80 and 100. The primary tumor location was mainly the head of the pancreas, in 70% of the enrolled patients. One patient presented lesions in both pancreas head and body. T3 and T4 tumors represented the majority of cases, 40% and 35% respectively.

**Table 1:** Patient and tumor characteristics

	Patients	%
**Sex**		
Female	9	45%
Male	11	55%
**Age**		
≤ 60 years	9	45%
> 60 year	11	55%
Median [min - max]	62 [44 - 74]	-
**Performance status**		
80%	7	35%
90%	7	35%
100%	6	30%
**Diagnostic method**		
Histologic	7	35%
Cytologic	13	65%

**Tumor differentiation**		
Well	7	35%
Moderately	1	5%
Poorly or not	6	30%
Missing	6	30%

TNM classification*		
T2N0	1	5%
T3N0	4	20%
T3N1	4	20%
T4N0	3	15%
T4N1	4	20%
Missing**	4	20%

**Localization**		
Head and body	1	5%
Head alone	14	70%
Body alone	4	20%
Tail alone	1	5%

Size of lesions (mm)***		
Primitive	39 [10 - 79]	
Primitive + lymph nodes	42 [10 - 79]	
**Total**	**20**	**100%**

*All tumors were M0

** Missing: 1 T3Nx (1), T4Nx (2) and TxNx (1)

*** Median size and range for the longest tumoral diameter, data missing for one patient

### Treatment Duration and Dose-Intensity

All 20 patients received chemotherapy and radiotherapy (CTRT).

### Chemotherapy

Median treatment duration was 6 cycles (range: [3 - 7]). Two patients received more than 130 mg/m^2 ^of DCT or 8750 mg/m^2 ^of 5-FU: one because he received one 1.8 Gy fraction of radiotherapy on week 7, the other one because of weight gain during treatment. Two patients received less than 120 mg/m^2^: one because of non hematologic toxicity (hyperglycemia during cycle 4, grade 4 vomiting and low blood pressure at cycle 6) and one received a decreased dose of DCT (106 mg/m^2^) for 6 weeks. The median relative dose-intensity was 99% of the theoretical dose (83%-106%) for DCT, and 98% (12%-106%) for 5-FU. Overall no treatment was stopped.

### Radiotherapy

The median number of treatment sessions was 30 [26 - 31]. Fifteen patients (75%) received the planned dose of radiotherapy, i.e. 54 Gy in 30 fractions. Only one deviation was observed with respect to the protocol with a case of non-toxicity-related prolongation of treatment where the patient received 31 fractions of 1.8 Gy, i.e. 55.8 Gy in total. Four patients (20%) received less than 54 Gy. Three patients received 28 fractions in 7 weeks, i.e. 50.4 Gy. Causes of treatment delay were mainly maintenance of radiotherapy machines and bank holidays. One patient suffered also from a pelvis fracture at week 4 and another experienced an unspecified toxicity at week 7. One patient received only 26 fractions in 6 weeks, i.e. 46.8 Gy. At week 6, he received only one instead of 5 fractions because of grade 4 vomiting and hypotension; 5-FU chemotherapy and radiotherapy were subsequently interrupted. Overall, the median number of administered cycles was 6; there were no interruptions of treatment, and the dose intensity was 98% of the theoretical dose. Median dose of radiotherapy was 54 Gy; only 3 patients received less than the theoretical dose.

### Toxicity

All patients were evaluated for adverse events (Table 2). Twelve patients (60%) experienced grade 3-4 toxicities during CTRT treatment. There were no treatment-related deaths. The most relevant severe toxicity involved the gastrointestinal tract in 17 patients (85%) including vomiting (35%), nausea (20%), abdominal pain (10%), anorexia (5%), diarrhea (5%), stomatitis (5%) and dyspepsia (5%). Grade 3 fatigue was observed in two patients. Grade 4 low blood pressure (1 pt) and gastrointestinal bleeding (1 pt) related to tumor progression occurred in two distinct patients. Five patients experienced grade 2 weight loss. At week 12 (i.e., 5 weeks after radiochemotherapy treatment completion), toxicities of any grades affected 16 patients (80%) and ranged from general (10 pts), hematologic (10 pts) and gastrointestinal (9 pts) for those with highest occurrence rates to hepatic (4 pts), cardiovascular (1 pt) and dermal (1 pt) for the less frequent ones. Most of them were grades 1 or 2. However, the following toxicities sorted by increasing severity are to be noted: grade 2 alopecia and cutaneous reaction of the hands and feet (1 pt); grade 3 hepatic toxicity (bilirubin 1 pt, GGT 1 pt) and grade 4 pulmonary emboli and cardiac complication (1 pt).

**Table 2:** Description of toxicities during CTRT

Type of toxicity	Grade 3 N (%)	Grade 4 N (%)
Vomiting	5 (25%)	2 (10%)
Fatigue	2 (10%)	-
Hypokalemia	2 (10%)	-
Anorexia	1 (5%)	-
Diarrhea	1 (5%)	-
Nausea	4 (20%)	-
Stomatitis	1 (5%)	-
Abdominal pain	1 (5%)	1 (5%)
Dyspepsia	1 (5%)	-
Hyperglycemia	1 (5%)	-
Deterioration of general condition	1 (5%)	-
Hypotension	-	1 (5%)
Gastrointestinal bleeding	-	1 (5%)

### Response and Survival

**Figure 2:**
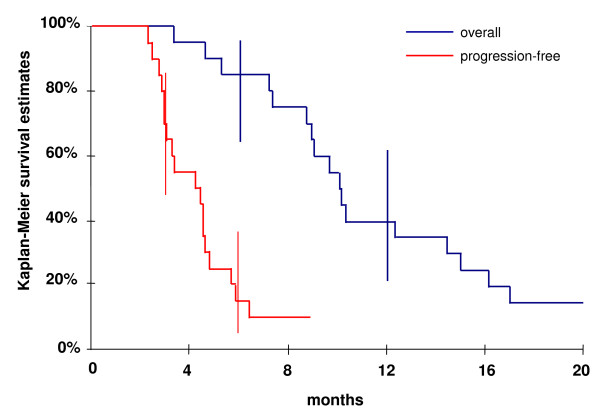
**Overall and progression-free survival**.

All 20 patients were assessable for response and survival. At 6 months, 18 patients (90%) had progressive disease. One year after week 12, the median duration of disease stabilization was 139 days (range: 125 - 378). The best response observed was complete response (CR) (1 pt, confirmed by surgery), partial response (PR) (1 pt, confirmed by a second evaluation) and disease stabilization (DS) (10 pts) with an objective response (2 confirmed responses) rate of 10% (95% CI, [0-23%]). The disease control rate, partial responses (2 pts) or stable disease (10 pts), measured at week 12 was 60% (95%CI, [38-81%]). No progression was observed during treatment. Nineteen patients (95%) died afterwards, all from their cancer within 17 months. Median overall survival time was 10 months (range: [3 - 69] months) (Figure 2). Six- and 12-month survival rate were 85% (95%CI, [64%-95%]) and 40% (95% CI [22%-61%]) respectively. Median progression-free survival (PFS) was 4 months (range: [2 - 69] months) (Figure 2). PFS percentages at 3 and 6 months were 70% (95% CI, [48% - 85%]) and 15% (95% CI, [5% - 36%]), respectively. One patient is still alive and has been in complete remission since he underwent tumor resection 66 months ago. Decision to stop inclusions in this study arm was subsequently made, due to the lack of treatment efficacy.

### Long-term survival with complete remission

We observed a long-term survival showing complete response with an absence of progression and no detectable disease for a period of 66 months following tumor resection. The patient, aged 55 years, was included in the treatment protocol less than a month after he was diagnosed as having pancreatic cancer. Ultrasound examination revealed a tumoral lesion of TNM grade US T3N0Mx located in the pancreas body with no peripheral adenopathy detected. Two biopsies were taken showing the presence of an exocrine pancreatic adenocarcinoma, and no criterion indicating the possibility of tumor surgical resection was observed.

The patient received 6 cycles of chemoradiotherapy in compliance with the trial's protocol. One month after the end of chemoradiotherapy, his tumor exhibited a partial response with a volume shrinking to 64% as well as a drop in CA 19-9 marker count from 3000 to 30. In regard to this remarkable result subsequent resective surgery was proposed.

Left splenopancreatectomy was performed with tumor margin-free en bloc resection of the pancreas body and tail and cholecystectomy for gallstones. The en-bloc resected piece was 10 cm long for 5 cm in diameter at the level of the resection margin. The result of the extemporaneous resection margin examination was suspect (presence of non-neoplasic dystrophic lesions) and two additional transverse cuts were necessary to obtain a free resected margin. The eleven lymph nodes found were all negative (11N-/11).

One year after surgery the patient had recovered from a weight loss of 15 kg. Sixty six (66) months after surgical resection of his tumor, the patient was still living and in good health (complete remission). The thorax-abdomen-pelvis CT-scans that are performed every 6 months did not reveal any specific sign of disease locoregional relapse or distant extension and the CA 19-9 markers remained permanently low.

## Discussion

In the face of the lack of progress observed during the past two decades in the curative-intent treatment of LAPC, the combined-modality treatment based on concurrent radiation therapy and chemotherapy remains the commonly accepted treatment and the sole prospect for improving disease outcome. However, the chemoradiotherapy optimal schedule and whether it should be administrated pre-operatively or post-operatively are yet to be determined.

One essential reason to investigate neoadjuvant chemoradiotherapy schedules relates to the possibility of gaining disease locoregional control in unresectable or borderline pancreatic cancer which represents a first step toward curative intent. In a recent review, the results of 13 phase II studies, published from year 2000 onwards, including 510 patients with unresectable LAPC treated by standard radiotherapy and concurrent chemotherapy were compiled [19]. This study has shown that resection rates ranged from 8 to 64% and among the operated patients, 57 to 100% (median, 87.5%) had tumor resection with negative margin (R0). Surprisingly, in patients with unresectable tumor at presentation, median survival after surgery ranged from 16.4 to 32.3 months as compared with 9 to 13 months for concurrent chemoradiation without surgery. Thus, pre-operative chemoradiation can play a beneficial role beyond palliative treatment of this type of tumor and the design of optimally successful schedules remains a relevant issue.

In our trial, 18 patients (90%) had progressive disease at the time of the intermediate analysis, with a median PFS of only 4.8 months, and a median survival of 10.1 months. These results are not better than those achieved with other treatment regimens for LAPC, including phase II trials that combined radiotherapy and chemotherapy [20-22]. In compliance with the pre-established continuation/discontinuation rules of the trial, this protocol treatment (docetaxel + 5-FU + RT) was not deemed efficient enough to justify further investigations and advance to phase III trial.

Pronounced toxicity side effects were observed during treatment (12 pts) and in week 12 (7 pts) with a majority of patients (75%) who have experienced grade 3-4 events, occurring predominantly in the gastrointestinal tract. Hematological toxicity was mild and non-hematological symptoms were similar to those previously reported including significant fatigue, lack of appetite, abdominal pain, nausea and emesis [23,24]. Overall, tolerance was comparable with other chemoradiotherapy regimens and would not have hampered the treatment feasibility if it had had the expected efficacy.

The concept that primarily unresectable pancreatic cancer are amenable to surgery is further supported by a number of published case reports, of dramatically advanced and/or metastatic primarily unresectable pancreatic cancer leading to surgical resection and 'long-term' survival [25-27]. In those reported cases long-term survival usually means survival prolonged for 1-3 years which already represents a substantial victory, especially in regards to the minimal expectations of the initial prognosis. For patients undergoing curative resection, the prognosis appears to be determined by tumor biology rather than factors involved in the resection [6,28,29]. The accurate proportion of complete remission, i.e. complete and permanent response without detectable signs of disease relapse, is to our knowledge currently not available, but seems to be extremely low (ca. 0.2% at 3 years) in histologically confirmed pancreatic carcinoma cases [30]. Further surgical and/or chemotherapeutic treatment of metastases or second cancer is very frequent (> 50%) in 5-year and longer survival cases also characterized by high comorbidity [4]. Long-term complete remissions such as the one we report here should be given more scientific attention and systematically collected for further re-examination to help establish better prognostic and/or treatment schedules, as they prove that there is hope even in this most dreadful pathology [31].

## Conclusions

Despite good tolerance, concurrent 5-FU-docetaxel radiotherapy cannot be recommended as a standard of care or even be tested in a subsequent phase III study. Inclusions have continued in the 5-FU-cisplatin arm, final results are awaited.

## Competing interests

Michel Ducreux has participated to advisory boards for Aventis during the accrual period of the study.

The (other) authors declare they have no competing interest."

## Authors' contributions

MD, PC, FV, and JPP conceived of the study and helped to draft the manuscript, JB and her team coordinated the study.

JPP, CB, LO and MD participated in data analysis. FV, JB, CB, MY, FM, LO, PC, MD, JB participated in data collection. All authors read and approved the final manuscript.
